# Cost Analysis of Inpatient Rehabilitation after Spinal Injury: A Retrospective Cohort Analysis

**DOI:** 10.7759/cureus.5747

**Published:** 2019-09-24

**Authors:** Austin Gamblin, Jason G Garry, Herschel W Wilde, Jared C Reese, Brandon Sherrod, Michael Karsy, Jian Guan, Janel Mortenson, Alexandra Flis, Jeffrey P Rosenbluth, Erica Bisson, Andrew Dailey

**Affiliations:** 1 Neurosurgery, Clinical Neurosciences Center, University of Utah, Salt Lake City, USA; 2 Neurosurgery, University of Utah School of Medicine, Salt Lake City, USA; 3 Physical Medicine and Rehabilitation, University of Utah, Salt Lake City, USA

**Keywords:** cost, spinal cord injury, value, value-driven outcome, sci, vdo, traumatic spine injury

## Abstract

Objective

The lifetime direct and indirect costs of spinal injury and spinal cord injury (SCI) increase as the severity of injury worsens. Despite the potential for substantial improvement in function with acute rehabilitation, the factors affecting its cost have not yet been evaluated. We used a proprietary hospital database to evaluate the direct costs of rehabilitation after spine injury.

Methods

A single-center, retrospective cohort cost analysis of patients with acute, traumatic spine injury treated at a tertiary facility from 2011 to 2017 was performed.

Results

In the 190 patients (mean age 46.1 ± 18.6 years, 76.3% males) identified, American Spinal Injury Association impairment scores on admission were 32.1% A, 14.7% B, 14.7% C, 33.2% D, and 1.1% E. Surgical treatment was performed in 179 (94.2%) cases. Most injuries were in the cervical spine (53.2%). A mean improvement of Functional Impairment Score of 30.7 ± 16.2 was seen after acute rehabilitation. Costs for care comprised facility (86.5%), pharmacy (9.2%), supplies (2.0%), laboratory (1.5%), and imaging (0.8%) categories. Injury level, injury severity, and prior inpatient surgical treatment did not affect the cost of rehabilitation. Higher injury severity (p = 0.0001, one-way ANOVA) and spinal level of injury (p = 0.001, one-way ANOVA) were associated with higher length of rehabilitation stay in univariate analysis. However, length of rehabilitation stay was the strongest independent predictor of higher-than-median cost (risk ratio = 1.56, 95% CI 1.21-2.0, p = 0.001) after adjusting for other factors.

Conclusions

Spine injury has a high upfront cost of care, with greater need for rehabilitation substantially affecting cost. Improving the efficacy of rehabilitation to reduce length of stay may be effective in reducing cost.

## Introduction

Spine fractures account for 3-6% of all skeletal fractures while acute spinal cord injury (SCI) has an estimated prevalence of 54 cases per 1 million people in the United States and an overall in-hospital mortality rate approaching 8% [1]. After initial stabilization and treatment, SCI patients typically require and benefit from intensive acute rehabilitation [2]. In the Veterans Healthcare Administration, each patient with SCI faces a lifetime healthcare cost of $1.1 to $5.4 million depending on the level of injury and patient age [3].

Inpatient rehabilitation after spine injury and SCI is a time- and resource-intensive endeavor. Well-trained and highly specialized teams of physiatrists, physical therapists, occupational therapists, and other medical professionals are required for optimum rehabilitation outcomes [2]. Many patients have co-occurring injuries such as traumatic brain injury, other polytrauma, and medical comorbidities, which can be associated with worse rehabilitation outcomes [4]. The level of therapy intensity and length of stay also have the potential to affect costs. The largest driver of overall health care costs for patients with traumatic SCI in one Canadian study was rehabilitation costs [5]. Importantly, the true hospital-level costs associated with acute inpatient rehabilitation for SCI patients, including breakdown of cost differences for different SCI severity, have not previously been evaluated. The purpose of this study was to evaluate the direct costs of inpatient rehabilitation after spine injury.

## Materials and methods

Patient inclusion

After receiving Institutional Review Board approval with a waiver of informed consent, a cross-sectional analysis of patients treated for spine injury or SCI from an internal database at the Department of Physical Medicine and Rehabilitation at our institution from January 2011 to December 2017 was performed. Patients were excluded if they were <18 years of age, if they underwent admission to rehabilitation for a chronic SCI, if they underwent treatment for a non-traumatic SCI mechanism, or if they lacked complete clinical, radiographic, and cost data. A manual chart review was performed to verify patients' inclusion and obtain demographic, clinical, and surgical data. Patients were cross-referenced in the institutional costs database to acquire subtotal costs.

Surgical procedures

Patients underwent a range of surgical approaches in the cervical, thoracic, or lumbar spine at an outside hospital or at our institution. Patients transferred after initial evaluation or surgical stabilization were considered transferred patients while those directly admitted to our institution were considered admitted patients.

Analysis

Demographic data included patient age and sex. Patient status before surgery was assessed with the American Society of Anesthesiologists (ASA) physical status system. The American Spinal Injury Association (ASIA) injury severity (AIS) score and injury level were identified from the clinical records. The Functional Independence Measure (FIM) was acquired from admission and discharge rehabilitation records. Length of stay (LOS) and discharge disposition were captured as well.

The institutional database is an electronic resource that reports direct costs, in lieu of patient/insurer charges [6-11]. Total cost and subcategory costs, including pharmacy, imaging, supplies and implants, laboratory, and facility costs, were analyzed. Physician professional fee was not available as a subcategory. Facility costs encompass the salaries of non-physician healthcare staff, as well as power, water, and administrative hospital cost. Actual dollar amounts are not reported as per agreement with the University. Subcategory cost is reported as a percentage of total cost. Mean percentage of total cost was generated as an alternative to presenting actual cost data. Costs were totaled for the entire cohort of patients, and the fraction of total cost contribution for each patient was calculated. Thus, means, standard deviations, patient total, and subgroup costs could be compared. The mean % of total costs may not total 100%. For subgroup cost contribution, each patient's subgroup cost was divided by the total. For these calculations, percentages will equal 100%. Continuous variables are reported as means and standard deviations and were analyzed by t-test. Noncontinuous variables were analyzed by Chi-squared test. Linear correlation was used to compare continuous variables while one-way analysis of variance with Tukey post-hoc comparison was performed to compare multiple continuous variables. A multivariable logistic regression was used to correlate factors with potential to cost more than the median total cost for the group of patients. Variables that had a p < 0.2 on univariable analysis were included in the multivariable analysis. We used the risk ratio (RR) to predict the cost of patient rehabilitation depending on clinical factors, level of injury, and severity of injury. SPSS V20.0 (IBM Corp., Armonk, NY) was used for statistical analysis with p < 0.05 considered significant.

## Results

A total of 190 patients treated from 2011 to 2017 were included for analysis. Patient demographics and admission characteristics are displayed in Table 1. AIS on admission were 32.1% A, 14.7% B, 14.7% C, 33.2% D, and 1.1% E. Surgical treatment was performed in 179 (94.2%) cases, with the majority of surgeries being performed at our institution. The majority of SCIs were localized to the cervical spine (54.2%), followed by thoracic (23.7%) and lumbar (15.3%) sites. The majority of patients were male (76.3%). Functional status was significantly improved for the SCI cohort as a whole when comparing pre-rehabilitation FIM scores vs. post-rehabilitation FIM scores (mean change +30 points in FIM score at discharge vs. admission).

**Table 1:** Demographics of 190 patients with spinal cord injury ASIA: American Spinal Injury Association; FIM: Functional Independence Measure.

Variable	Value
Mean age (±STD), years	46.1 ± 18.6
Sex (%), male	145 (76.3%)
Injury level	
Cervical	103 (54.2%)
Thoracic	45 (23.7%)
Lumbar	29 (15.3%)
Sacral	2 (1.1%)
Cauda equina	9 (4.7%)
Unknown	2 (1.1%)
Surgical treatment (%)	
None	9 (4.8%)
Tertiary facility	127 (66.8%)
Other facility	52 (27.4%)
Unknown	2 (1.1%)
Multiple rehabilitation admissions	15 (7.9%)
Mean time from injury to rehabilitation (±STD), days	17.9 ± 42.4
Mean time from injury to surgery (±STD), days	1.8 ± 4.0
Mean rehabilitation length of stay (±STD), day	32.9 ± 23.1
Mean follow-up (±STD), days	24.2 ± 25.6
ASIA impairment score	
A	61 (32.1%)
B	28 (14.7%)
C	28 (14.7%)
D	63 (33.2%)
E	2 (1.1%)
Unknown	8 (4.2%)
Mean FIM score (±STD)	
Admission	56.2 ± 18.7
Discharge	86.9 ± 25.6
Change	30.7 ± 16.2
Post-rehabilitation disposition (%)	
Acute rehabilitation	4 (2.1%)
Other hospital	16 (8.4%)
Home	60 (31.6%)
Home health	36 (18.9%)
Skilled nursing facility	24 (12.6%)
Unknown	50 (26.3%)

A breakdown of surgical procedures by site shown in Table 2 demonstrated that a variety of anterior, posterior, or combination procedures were performed depending on injury level.

**Table 2:** Surgical treatment of 190 patients with spine injury ACDF: Anterior cervical discectomy and fusion

Surgery type	Number of cases
Cervical, n = 103 (54.2%)	
2-level ACDF	18
2-level posterior fusion	8
2-level ACDF and 2-level posterior fusion	2
2-level ACDF and 4-level posterior fusion	1
3-level ACDF	9
3-level posterior	13
3-level ACDF and 2-level posterior fusion	1
3-level ACDF and 3-level posterior fusion	2
3-level ACDF and 5-level posterior fusion	1
3-level ACDF and 6-level posterior fusion	1
4-level ACDF	2
4-level ACDF and 4-level posterior fusion	1
4-level posterior fusion	7
5-level ACDF	1
5-level posterior fusion	9
6-level posterior fusion	9
7-level posterior fusion	1
8-level posterior fusion	2
9-level posterior fusion	1
Decompression alone	7
Fragment removal	1
No surgery	4
Unknown	2
Thoracic, n = 45 (23.7%)	
2-level anterior fusion	1
3-level anterior and 7-level posterior fusion	1
3-level posterior fusion	11
4-level posterior fusion	2
5-level posterior fusion	9
6-level posterior fusion	8
7-level posterior fusion	4
9-level posterior fusion	1
10-level posterior fusion	2
Decompression alone	2
No surgery	2
Vertebroplasty and decompression	1
Unknown	1
Lumbar, n = 29 (15.3%)	
2-level posterior fusion	1
3-level posterior fusion	9
4-level posterior fusion	4
5-level posterior fusion	6
7-level posterior fusion	1
Decompression alone	3
No surgery	1
Vertebroplasty	1
Unknown	3
Sacral, n = 2 (1.1%)	
Sacral fixation	1
No surgery	1
Cauda equina, n = 9 (4.7%)	
3-level anterior fusion	1
3-level posterior fusion	2
4-level posterior fusion	1
5-level posterior fusion	2
7-level posterior fusion	1
Decompression	1
No surgery	1
Unknown, n = 2 (1.1%)	

**Figure 1:**
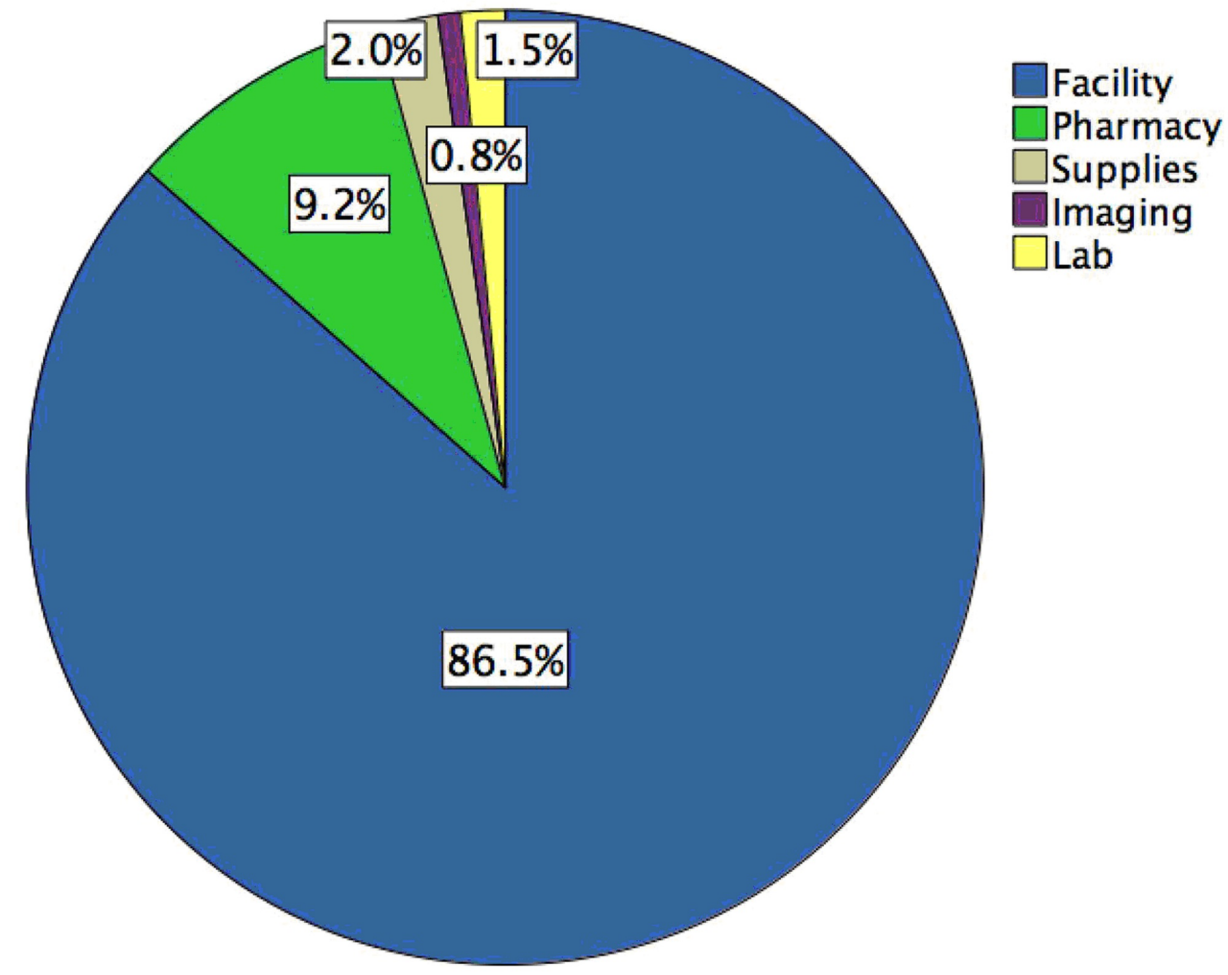
Cost distribution for acute rehabilitation of spine injury patients

A graphical breakdown of total rehabilitation costs is displayed in Figure 1. The vast majority of overall rehabilitation cost was attributable to facility costs (90%), followed by pharmacy costs (9%), with the remaining percentage attributable to supplies, imaging, and laboratory costs. Facility costs incorporate the costs of therapy and nursing in addition to other facility management costs.

Table 3 shows the results from the logistic regression cost predictor analyses (both univariate and multivariate). In the univariate analysis, rehabilitation LOS (unadjusted RR 1.44, 95% CI 1.2-1.7, p = 0.0001) and ASIA B severity (unadjusted RR 12.75, 95% CI 1.26-128.7, p = 0.03) were the only variables significantly associated with higher rehabilitation costs. No significant correlations were seen between cost and age, sex, surgical treatment, time from injury to surgery, time from injury to rehabilitation, or FIM score change. In the multivariate analysis, rehabilitation LOS was the only independent predictor of greater rehabilitation cost (adjusted RR 1.56, 95% CI 1.21-2.0, p = 0.001).

**Table 3:** Cost drivers in the care of spinal cord injury rehabilitation ASIA: American Spinal Injury Association; FIM: Functional Independence Measure

	Univariate			Multivariate		
Variable	Odds ratio	95% CI	P-value	Odds ratio	95% CI	P-value
Age	0.999	0.981, 1.17	0.9			
Sex						
Female	1.16	0.54, 2.48	0.7			
Male	Reference					
Injury level						
Cervical	3.26	0.56, 19.02	0.2	16.2	0, -	0.8
Thoracic	2.34	0.36, 13.78	0.4	3.0	0, -	0.9
Lumbar	0.38	0.05, 3.06	0.4	1.4	0, -	0.98
Sacral	-	-	-	-	-	-
Cauda equina	Reference			Reference		
Surgical treatment						
None	Reference					
Tertiary facility	0.31	0.06, 1.69	0.2			
Other facility	0.57	0.1, 3.24	0.5			
Multiple rehabilitation admissions	0.82	0.24, 2.82	0.8			
Time from injury to rehab	0.998	0.99, 1.005	0.6			
Time from injury to surgery	0.97	0.89, 1.07	0.6			
Rehabilitation length of stay	1.44	1.2, 1.7	0.0001	1.56	1.21, 2.0	0.001
ASIA impairment score						
A	3.00	0.46, 19.8	0.3	-	-	1.0
B	12.75	1.26, 128.78	0.03	-	-	1.0
C	2.33	0.32, 16.82	0.4	-	-	1.0
D	0.19	0.03, 1.45	0.1	-	-	1.0
E	Reference	-	-	Reference		
FIM score change	0.99	0.98, 1.02	0.9			

**Figure 2:**
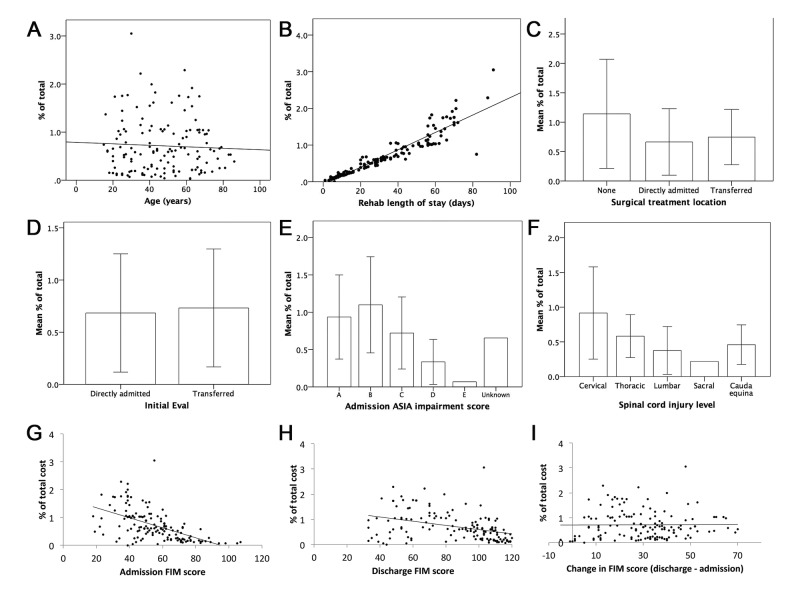
Subgroup analysis of potential cost drivers during acute rehabilitation of spine injury patients The Y axes represent % of total cost for the entire cohort, with each patient's individual cost contributing to that total (represented as points in A, B, G–I) and as mean ± STD (C–F). This strategy allows comparison of patient costs without reporting direct dollar amounts. Potential cost drivers were (A) age; (B) rehabilitation length of stay; (C) site of surgical treatment; (D) type of admission; (E) ASIA score; (F) SCI level; (G) admission FIM score; (H) discharge FIM score; and (I) change in FIM score. ASIA: American Spinal Injury Association; SCI: Spinal cord injury; FIM: Functional Independence Measure.

Subgroup analyses for individual variable contributions to overall costs are displayed graphically in Figure 2. The Y axes represent % of total cost for the entire cohort, with each patient's individual cost contributing to that total (represented as points in panels A, B, G, H, and I, and as mean ± standard deviation cost for panels C-F). Figure 2A shows that there was no correlation between age and % individual contribution to total rehabilitation cost (β = -0.002, p = 6), whereas Figure 2B demonstrates the strong direct trend for LOS and % individual contribution to total rehabilitation cost (β = 0.024, p = 0.0001). There were no significant differences among surgical treatment location (p = 0.09) or transfer/direct admission status (p = 0.06) for mean individual contribution to % of total costs (Figure 2C, 2D). Differences in mean individual % of total costs related to spine injury severity indicated that patients with ASIA grades B and A incurred higher costs (p = 0.0001), with ASIA B costing more than ASIA D (p = 0.0001, Figure 2E). Higher AIS severity did show a significantly longer LOS (p = 0.0001, one-way ANOVA). LOS of 44 ± 20, 47 ± 25, 33 ± 22, 17 ± 13, and 9 ± 6 days were seen for ASIA grade A, B, C, D, and E patients, respectively. Similarly, there were higher mean individual costs for cervical spine and thoracic injury location vs. lumbar cases (p = 0.0001, Figure 2F). Higher injury levels also showed a significantly longer LOS (p = 0.001, one-way ANOVA). LOS of 39 ± 26, 30 ± 15, 20 ± 15, 13, 22 ± 15 days were seen for cervical, thoracic, lumbar, sacral, and cauda equina levels, respectively. Admission FIM correlated with cost (β = 1.71, p = 0.0001), although this was not significant on logistic regression analysis (Figure 2G). Figure 2H and Figure 2I show that there was no obvious trend indicating a correlation of discharge FIM score (β = -0.008, p = 0.0001) or change in FIM score (β = 0.0001, p = 0.9) with individual cost contribution.

## Discussion

Overall, our results suggest that rehabilitation LOS impacts costs of rehabilitation most significantly after acute, traumatic spine injury. Strategies to improve the efficiency of treatment and reduce LOS would have the greatest cost savings for the spine injury population. In addition, injury severity and level suggested longer LOS but this did not correlate with higher cost in every case. In addition, less severe injury may allow for more rapid patient recovery and transition to home or outpatient services. Higher LOS was seen for ASIA grade B lesions compared with other groups, suggesting strategies to maximize rehabilitation in this patient group may have the biggest effect on controlling costs. Variability in cost was seen for each AIS grade and level of injury, which may suggest that perhaps other factors can account for some cost variation among patients. Understanding this variability and potentially streamlining care may produce some cost savings.

Healthcare costs are a significant burden to society, with 2016 annual healthcare expenditures reaching nearly 20% of the United States gross domestic product [12]. Although pharmaceutical costs have been implicated as the primary driver for increases in healthcare-related spending, nearly all aspects of patient care have become more costly over the last decade [13]. Within the SCI literature, Selvarajah et al. reported data from the Nationwide Emergency Department sample years 2007-2009, finding that $4.8 billion (in 2009 dollars) in hospital charges accumulated for traumatic SCI patients [14]. One 2007 study of SCI patients at 3 Veteran's Health Administration hospitals showed that direct cost per SCI patient amounted to $21,450, with complete cervical SCI being most costly and incomplete thoracic cervical SCI being least costly [3]. Munce et al. found that inpatient rehabilitation costs were the largest driver of total direct costs for SCI patients at a single center in Ontario, accounting for 58% of overall cost [5]. However, none of these studies reported a breakdown of healthcare costs specifically associated with rehabilitation in the SCI population.

There were several pertinent findings in the current study. Facility costs accounted for nearly 90% of the total rehabilitation costs in our study. When compared with other healthcare settings, inpatient rehabilitation typically requires fewer pharmaceutical interventions and laboratory studies and instead requires more facility resources for rehabilitation activities (e.g., physical therapy, recreation), which may explain the relative contribution of facility costs versus pharmacy and other costs. However, much of the post-hospitalization care of patients with spine injury, including medications and other services, continue during rehabilitation. Nonetheless, the acute rehabilitation by and large covers the largest component of cost. Previous studies by our group also suggest that, in cases that instrumentation or devices are not heavily used (e.g., spine, endovascular), facility costs contribute most to overall cost [7]. No significant rehabilitation cost difference was observed among different injury severity ASIA classifications or anatomic spinal locations. This was a surprising finding given that ASIA classification is a measure of severity, and we hypothesized that injury severity would directly correlate with rehabilitation costs. Although there was a trend toward higher cost with greater injury severity (Figure 2E), the difference was not significant after adjusting for length of rehabilitation stay. Higher injury severity and levels did result in longer LOS, but there remained significant variation among severity and levels in terms of cost. One potential limitation is that this comparison may have been underpowered statistically. Prior studies have shown that SCI patients with lower injury severity spent fewer days in the hospital and had lower overall costs of inpatient care [15]. Our findings are similar but showed that LOS was a predominant cost driver even after controlling for level and severity of injury. Improving cost may be potentially seen by standardizing treatments to reduce variability. In addition, planning transitions of care for patients with more significant injury could reduce the length of time necessary to improve the discharge readiness of patients.

Study limitations

This study is not without limitations. A single-center analysis limits analysis of rehabilitation costs across different health systems. Therefore, the results presented herein may not be generalizable across different rehabilitation facilities. Furthermore, our study was performed at an academic rehabilitation center and may not be reflective of costs in other practice models (e.g., in the private sector). The study population did however reflect the expected distribution of age, sex, and AIS injury grade found nationally [16]. Our study cohort, although large for a rehabilitation cohort, is still relatively small and may be underpowered to capture the full clinical spectrum and heterogeneity that exists among patients with spine injury. The direct correlation between rehabilitation and eventual patient outcome, as well as selection of rehabilitation duration, was not completely clear. Further prospective study, and adjustment for these variables, would be needed to better understand the impact of rehabilitation on patient outcomes. The institutional database has its own limitations as a data source. Physician professional fees are not available as a cost variable, and actual dollar amounts are not reported as per agreement with the University. The inability to analyze actual dollar amounts limits our analysis to relative analysis only (i.e., we can only compare costs vs. other injured patients and cannot compare our center's costs with those from another rehabilitation center). We also are unable to obtain the indirect costs of care for patients, which is likely quite substantial for this patient population. Despite these limitations, this study adds insight into the direct costs associated with inpatient rehabilitation after spine injury.

## Conclusions

We present data on our institution's experience with direct costs of acute inpatient rehabilitation after spine injury. Facility costs accounted for the vast majority of rehabilitation costs. Length of stay was the only independent predictor of increased rehabilitation costs. Spine injury continues to have a high upfront cost of care, with the great need for rehabilitation playing a key role in cost. Improving the efficacy of rehabilitation in order to reduce length of stay and streamline care may be important in reducing costs.
